# Gut indigenous microbiota and epigenetics

**DOI:** 10.3402/mehd.v23i0.17195

**Published:** 2012-03-28

**Authors:** Boris Arkadievich Shenderov

**Affiliations:** Laboratory of Biology of bifidobacteria, Head of Research Group Probiotics and Functional Foods, Gabrichevsky Research Institute of Epidemiology and Microbiology, Moscow, Russia

**Keywords:** gut microflora, microbial small molecules, mammalian epigenomics

## Abstract

This review introduces and discusses data regarding fundamental and applied investigations in mammalian epigenomics and gut microbiota received over the last 10 years. Analysis of these data enabled us first to come to the conclusion that the multiple low-molecular-weight substances of indigenous gut microbiota origin should be considered one of the main endogenous factors actively participating in epigenomic mechanisms that are responsible for the mammalian genome reprograming and post-translated modifications. Gut microecological imbalance caused by various biogenic and abiogenic agents and factors can produce different epigenetic abnormalities and the onset and progression of metabolic diseases associated. The authors substantiate the necessity to create an international project ‘Human Gut Microbiota and Epigenomics’ that facilitates interdisciplinary collaborations among scientists and clinicians engaged in host microbial ecology, nutrition, metagenomics, epigenomics, and metabolomics investigations as well as in disease prevention and treatment. Some priority scientific and applied directions in the current omic technologies coupled with gnotobiological approaches are suggested that can open a new era in characterizing the role of the symbiotic microbiota small metabolic and signal molecules in the host epigenomics. Although the discussed subject is only at an early stage its validation can open novel approaches in drug discovery studies.

For many decades, it has been thought that health and diseases are predominantly driven by acquired genetic changes. Now, it is becoming clear that any phenotype is the result of complex interactions between genotype, epigenome, and environment. Epigenetics focuses on processes that regulate how and when certain genes are turned on and turned off, while epigenomics analyzes epigenetic changes across many genes in a cell or entire organism. In the recent years epigenetics is considered to be at the epicenter of modern medicine because it helps to explain the relationship between individual genotype and the environment during all stages of living beings and perturbations in epigenetic mechanisms can result in various health disturbances (1–3). Epigenomic reprograming of cell genome and posttranslation modification of gene expression are essential mechanisms of the development, regeneration, and postnatal life of higher eukaryotic organisms (gene expression regulation, cell proliferation, cellular stress events, aging and DNA repair, lifelong circadian drifts, equilibrium between mitosis and apoptosis, modification of bacterial and host cell quorum sensing, host–bacteria cross-talk, and so on) (3, 4) as well as in the bacterial world (gene regulation, virulence of human and animal pathogens, timing of DNA replication, repair of DNA, phase variation, and so on) (5). Human epigenetics may explain some features of various monogenic and multifactorial disorders (metabolic syndrome, type II diabetes, schizophrenia, autoimmune diseases, cancer, autism, and so on) as well as their late onset, gender effects, fluctuation of symptoms, phenotypic differences between monozygotic twins and others. Epigenetic code can be individual as well as tissue- and cell-specific and may change over time because of aging, disease, or environmental factors and agents. Epigenetics does not involve an alteration in the nucleotide sequence of the DNA; epigenomic effects are connected with the covalent attachment of different chemical groups to DNA, chromatin, histones, and other associated proteins during post-translation period. Examples include DNA and histone methylation, acetylation, biotinylation, phosphorylation, ADPribosylation, repeat-induced gene silencing, miRNA interferences, ubiquitination, sumoylation, genomic imprinting, and so on. Epigenetic DNA and chromatin alterations persist from one cell division to the next and can occur for several cell generations (1–3). Determination in the body fluids methylated DNA, acetylated proteins, miRNA, specific substrates, cofactors, enzymes participating in the biochemical reactions connected with epigenomics processes could be biomarkers for detecting some metabolic diseases (3, 6, 7).

Now it is proved that a number of food ingredients, drugs, and environmental chemical pollutants can interfere into epigenomic gene regulation and post-translation modification of gene products (3, 8). The data also accumulated that certain infectious agents (*Epstein–Barr* virus, hepatitis viruses B and C, Human papilloma virus, polyomaviruses, *Streptococcus bovis, Chlamydia pneumoniae, Campylobacter rectus, Helicobacter pylori*, and so on) can contribute to the host epigenetic changes resulting in the onset and progression of some metabolic connected diseases, especially in malignancies (9, 10). The symbiotic microorganisms have been implicated in epigenetic thermotolerance variation of coral reef cnidarians symbiosis, pea aphids, and cactuses. (11). A role of mammalian gut microbiota, as epigenetic modifying factor, in the pathogenesis of metabolic syndrome and associated diseases has been meant (12, 13). Thus, the different biotic and abiotic signals can produce changes in gene expression that can persist after effect has ceased (14).

However, the epigenomic reprograming and post-translated molecular mechanisms connected with symbiotic microflora remain unclear. Through the analysis of different microbial molecules we will attempt to evaluate the role of gut indigenous microbiota in epigenomic mechanisms and the consequences of gut microecological imbalance and epigenetic abnormalities in the onset and progression of metabolic diseases associated.

## Microbe structural components and metabolites as potential epigenomic modifiers

The mammalian gastrointestinal tract is populated with as many as 100 trillion bacterial cells. Among the human gut microbiota there are health-promoting indigenous species that have the intriguing diversity and produce extremely important contributions to human physiology, biochemistry, behavior, metabolic signaling, metabolism of endogenous and exogenous compounds, stability of prokaryotic and eukaryotic DNA, its transcription and translation, as well as gene regulation of the microbial and eukaryotic cells expression (15–18). Recent omic-based studies have permitted to provide insights into how indigenous microbiota (including probiotics) sense and adapt to the gastrointestinal tract environment and regulate gene expression and post-translation modification of gene-determined final products in and outside the host intestinal tract (18–23). It is well known that indigenous microbiota produce multiple low-molecularweight (LMW) substances that can quickly be distributed along human organisms and interact with different targets in the cells, tissue, organs, and organism on the whole. Many of them are able to interfere in the genomic, epigenomic processes and in the host metabolism. Some of the microbial LMW molecules may be one of the key endogenous environmental factors regulating human genes expression throughout life (16, 18) (Table 1).

**Table 1:** Some gut microbiota-derived LMW molecules as potential inductors, participants, and modifiers of host epigenomic alterations

Proteins, peptides, polysaccharides, endotoxins, lectines Simple biochemical groups (methyl-, acetyl-) and compounds (biotin, betaine, methionine, lysine, arginine, serin, threonine, acetate, butyrate, propionate, adenosine, cytosine) Various enzymes (methyltransferases, acetyltransferase, deacetylases, BirA ligase, phosphotranferases, kinases, synthetases), Co-factors (folic acid, B12, B6, B2, choline, nicotinic acid, NAD, Coenzyme A) and signal molecules (hormone-like substances, inositol triphosphate), Activators and inhibitors of activity of enzymes participating in epigenomic regulation (butyrate, propionate, spermidine, sulforaphane cysteine)

The intensity of microbial signal and the subsequent gene response can vary with the composition and the number of certain indigenous intestinal microorganisms. The physiological condition of the individual also may determine which human genes could be under epigenomic influence of indigenous gut microbiota that serves as a source of different chemical groups, amino acid residues, modified bases, miRNA and long RNA, other metabolic and signaling molecules, enzymes or their co-factors participating in the epigenomic processes.

## Targets and some mechanisms of epigenomic modulation by indigenous microbiota LMW-bioactives

Microbial LMW molecules may alter epigenetic homeostasis by direct or indirect mechanisms. For example, production of bacterial methyl or acetyl groups, or biotin and methyltransferases, acetylase/deacetylase, or BirA ligase may directly affect chromatin architecture orDNA methylation, histone acetylation or other epigenetic mechanisms.

DNA from most prokaryotes and eukaryotes contains the methylated bases 4-methylcytosine, 5-methylcytosine, and 6-methyladenine. Modifications by methylation are introduced after DNA replication by DNA methyltransferases. Methylation is an enzymatic process in which covalent modification of the cytosine and adenine, and histone modifications occur, when several arginine and lysine residues in the N-termini of histones are methylated by various types of methyltransferases; a number of demethylases mediate the removal of methyl groups (24). Today, about 50–100 different methyltransferases have been identified in animal, plant, and microbial cells (25), including more than 20 lysine and 10 arginine histone methyltransferases (26). Lysine acetylation is also considered as one fundamental post-translational modification exerting effects on chromatin dynamics and other cellular processes. This modification transfers the acetyl moiety from acetyl-CoA to the ogroup of a lysine residue and is dynamically governed by two groups of counteracting enzymes known as lysine acetyltransferases (KATs) and NAD-dependent protein deacetylases (HDACs). Acetylation of specific lysine residues on histone is generally associated with transcriptional activation, where histone deacetylation results in transcriptional repression. In addition to histones, numerous non-histone proteins with various functions possess acetyl-lysine residues and may be substrates of KATs and HDACs. Regulation by lysine acetylation may operate at the gene expression level, enzymatic activity and/or protein stability (27–29). Methylation and demethylation (24), acetylation and deacetylation (27–29) are reversible and can be involved in both gene activation and silencing in a wide variety of prokaryotes and eukaryotes. There are data that some representatives of these both groups of enzymes, functionally and partly structurally, are very similar in eukaryotic and prokaryotic cells (28, 30, 31). Mammalian DNA methyltransferases function to change the methylation profile for specific compartments of the genome in a tissue-specific manner. In contrast, prokaryotic methyltransferases modify all of their recognition sites (9). Biotinylation is a epigenomic process that is characterized by the attachment of vitamin biotin to histone proteins resulting in gene repression. Mammalian cells cannot synthesize biotin and depend on a constant supply of food or intestinal microbiota biotin to maintain normal levels of protein biotinylation (32, 33). Biotin deficiency causes abnormally low biotinylation of histones and results in an aberrant gene regulation (e.g. derepression of retrotransposons leading to chromosomal instability) (34). Biotinylation of histone in mammalian cells is mediated by eukaryotic holocarboxylase synthetase (HCS), biotinidase and microbial non-selective enzyme (BirA ligase). BirA ligase plays a key role in the cell signaling and chromatin remodeling during biotin biosynthesis in prokaryotes. Similar mechanisms of gene regulation for HCS have been reported in eukaryotes (35). RNA-interference is an important epigenomic process in which specific genes can be turned off or silenced via mechanism mediated by a class of endogenous small RNA molecules of the same size in plants, worms, flies, mice, and humans. This small RNA (short – about 20, and long – about 150 nucleotides in length) was called miRNA. Double-stranded RNA is more effective in producing interference than was either strand individually (36, 37). Today, it is estimated that there are about 5000 miRNAs in mammalian cells, and that about 30% of all genes are regulated by miRNAs. The miRNAs can regulate gene expression by base-pairing to miRNA, which results in either degradation of the miRNA or suppression of their translation. In recent years, similar small RNA molecules were also revealed in prokaryotic organisms. For instance, about 25 cases of regulatory trans-acting antisense RNAs are known in *E.coli* (38).

An example of an indirectly acting of microbial LMW molecules to chromatin remodeling is the deficiency of some substrates (methionine, betaine, and choline) and/or cofactors (folate, vitamins B12, B2, and B6) produced by indigenous intestinal microbiota (16, 23, 39, 40). Being a methyl donor (or cofactors), all above-mentioned substances participate in the one-carbon pathway. Gut bacteria also affect the bioavailability of many dietary sources of methyl groups (41). Inadequate dietary and/or microbe methyl group and cofactor provision alters onecarbon metabolism and leads to hypomethylation in many important epigenomic-associated pathways. This alteration can impair DNA methylation resulting in elevated plasma homocysteine concentrations, decreased S-adenosylmethionine (SAM) content, and increased risk of various coronary, cerebral, hepatic, vascular diseases, and malignancy (16, 25, 42).

Chromatin remodeling has also been linked with the levels of total caloric intake. Energy sensing takes place, in part via the reduced ratio of NADH (nicotinamide adenine dinucleotide) to NAD+ (nicotinamide adenine nucleotide). An NAD + dependent histone deacetylase (sirtuin) appears to be a key modifier of chromatin structure. Acetylation and deacetylation of chromosomal histone proteins alter their interaction causing changes in gene expression regulation. The main donor of acetyl groups for formation of acetyl-CoA that participates in epigenomic acetylation reactions is gut microbiota. Bacteria/eukaryotes share a common pathway for coenzyme A (CoA) biosynthesis from pantothenate (vitamin B5), cysteine, and β-alanine. These essential cofactors are found in most foods in small quantities and are also generated by endogenously and/or by various gut microorganisms. Deficiencies in these nutrients of dietary or microbial origin impair synthesis of acetyl-CoA, NADH and NAD, resulting in disorders of epigenomic acetylation machine that is responsible for chromatin remodeling and post-translation modification of proteins (18, 23, 43). Another example of an indirect effect of indigenous microorganisms to human epigenome could be gut microbiota contributions to the transformation, bio-viability, absorption and/or excretion of some chemical elements (zinc, iodine, selenium, cobalt, and others), which are co-factors of various enzymes participating in the work of different epigenomic processes (13, 44). Besides, gut microbiota may contribute to the metabolism of different plant micronutrients (e.g. different phenolic compounds); microbe-derived metabolites may become important mediators interfering in the host metabolism, genomic and epigenomic processes (45, 46).

Some LMW molecules of microbial origin can directly or indirectly activate or inhibit epigenomic regulation through interference to activity of enzymes (methyltransferases, deacetylases, acetyltransferases, phosphotranferases, BirA ligase, various synthetases, nucleases, serine-threonine protein kinases and, so on), which are participants in epigenomic reprograming and/or posttranslation modification of histone and other proteins. As an example, short-chain fatty acids are normally produced by gut microflora, and related products have been approved for human use (16, 23, 43). Butyrate and propionate can inhibit histone deacetylase enzymes and alter the expression of specific genes via the conformation changes in the active site of HDAC resulting in its inactivation (47, 48). Microbe-derived butyrate, at physiological concentrations, can trigger cell cycle arrest and apoptosis in colon cell line through interference in the regulation of host gene expression via colonic epithelial HDAC inhibition (49, 50) or decreased expression of the miRNAs (miR-106b family) (15). These results are important for the understanding of intestinal homeostasis and carcinogenesis mechanisms.

During microbial metabolism of cruciferous vegetables or garlic a corresponding number of sulforaphane cysteine/sulforaphane N-acetyl-cysteine and allyl mercaptan/diallyl disulfide are formed that can inhibit the activity of histone deacethyltransferases enzymes (27). Searching for natural dietary and indigenous microbial modifiers of methyltransferases and other enzymes participating in the epigenomics gene regulation and post-translation modification is still emerging, especially with regard to personal drug, metabiotic, and food discovery (21, 26, 51–53).

A vast majority of reports on cross talk between bacteria and epithelial cells have focused on single bacterial strains. But in real life mammalian gut bacteria have never acted on the host cells in isolation, and about 1000 microbial phylotypes and tens of thousands of phylogenetic different bacterial strains in the adult human colon are engaged in constant cross talk with intestinal epithelial cells (39). This fact is necessary when we discuss the role of indigenous gut microbiota in the epigenetic phenomena.

Multiple LMW metabolites and signal molecules produced by indigenous gut and vaginal microbiota of pregnant women can penetrate via placenta into fetus, resulting in permanent effects of its development programing, cognitive function, metabolism, and body composition in the natal and postnatal periods of life through epigenomic activation or suppression of gene expression, or turning genes ‘on’ or ‘off ’. Information presented above has permitted to suppose that significant alterations in maternal indigenous microbiota may induce long-term metabolic consequences in offspring as a result of disorders of development programing. Thus, imbalance in the mother indigenous gut microbiota may affect adult health and life span as a result of epigenetic variations that may be established during embryonic and fetal development because of consequence of incomplete work of epigenetic machines. Increased supplementation of pregnant women with corresponding diet or microbial origin bioactives (enzymes, relevant cofactors, or their precursors) (54) and/or restoration of women gut microbiota with probiotics, prebiotics, or metabiotics will rehabilitate intracellular concentration of metabolic or signal molecules necessary for epigenomic modulation of DNA, chromatin, and histones or alteration of posttranslated final products (21).

## Conclusion

Thus, according to my opinion, diet nutrients and LMW molecules of indigenous microbiota with different metabolic and signal activity can be considered to be correspondingly the most significant exogenous and endogenous environmental determinants, that if not the key determinants, affect gene expression in the host metagenome and post-translation modification of gene products via various epigenetic biochemical mechanisms. These observations can become the basis for development of novel approaches in designing new medicines and diagnostic tests for different pathological syndromes having epigenomic components.

To date several large consortiums or initiatives have been found for investigation in the field of Human being Epigenome: The Human Epigenome Project in Europe; The Alliance for the Human Epigenome and Disease (AHEAD) formed by the American Association for Cancer Research Epigenome Task Force and the European Union Network of Excellence Scientific Advisory Board. In January 2008, The United States National Institute of Health announced that it would invest more than $190 million for the next five years in epigenomics biomedical research [www.neb.com/nebe4comm/tech_reference/epigenetics/epigenetics.asp; www.epi.grants.cancer.gov/epigen.html]. To help coordinate efforts, resources, and funding, the authors consider that it is necessary to fund the International Project ‘Human Gut Microbiota and Epigenomics (Microecological Epigenomics)’. The goals of this project will be to facilitate interdisciplinary collaborations among scientists and clinicians engaged in host microbial ecology, nutrition, metagenomics, epigenomics, as well as disease prevention and treatment. The main aim of such investigations is to establish the profile of microbial LMW substances that characterize the role of human indigenous microbiota in the epigenomic regulation of genome and microbiome activity and protein post-translation modification. The mid term goal is to provide metabolic databases that could be used for selection of microbe-associated biologically active LMW molecules participating in the epigenomic processes. Such small bioactive molecules, either extracted from indigenous (probiotic) microorganisms or their cultural fluids, or synthesized by biochemists, may be later used for activation or inhibition of specific signaling pathways/ enzymatic activities connected with epigenomic regulation effects. Humans are not good experimental subjects because every individual has a unique metogenoepigenotype and it is difficult to control for environmental influences. Therefore, the realization of any Epigenome Projects compulsorily requires active involvement of various gnotobiological models. Advances in the current omic-technologies, coupled with gnotobiological approaches, open a new era in characterizing the role of the symbiotic microbiota in the host epigenomics and have far-reaching implications in the conception relevant to human health and metabolic diseases. Understanding how small molecules of microbial origin interact with the epigenome will permit to design a new generation of epigenetic drugs for curing complex diseases such as cancer and metabolic syndrome-associated diseases.
